# Differential Reward Learning for Self and Others Predicts Self-Reported Altruism

**DOI:** 10.1371/journal.pone.0107621

**Published:** 2014-09-12

**Authors:** Youngbin Kwak, John Pearson, Scott A. Huettel

**Affiliations:** 1 Department of Psychological and Brain Sciences, University of Massachusetts Amherst, Amherst, Massachusetts, United States of America; 2 Center for Cognitive Neuroscience, Duke University, Durham, North Carolina, United States of America; 3 Duke Center for Interdisciplinary Decision Sciences, Duke University, Durham, North Carolina, United States of America; 4 Department of Psychology and Neuroscience, Duke University, Durham, North Carolina, United States of America; Inserm, France

## Abstract

In social environments, decisions not only determine rewards for oneself but also for others. However, individual differences in pro-social behaviors have been typically studied through self-report. We developed a decision-making paradigm in which participants chose from card decks with differing rewards for themselves and charity; some decks gave similar rewards to both, while others gave higher rewards for one or the other. We used a reinforcement-learning model that estimated each participant's relative weighting of self versus charity reward. As shown both in choices and model parameters, individuals who showed relatively better learning of rewards for charity – compared to themselves – were more likely to engage in pro-social behavior outside of a laboratory setting indicated by self-report. Overall rates of reward learning, however, did not predict individual differences in pro-social tendencies. These results support the idea that biases toward learning about social rewards are associated with one's altruistic tendencies.

## Introduction

The cardinal assumption of models of decision making – both in economics and psychology – is that individuals learn to make choices that maximize personal utility [1], [2]. Such models do not require that individuals consciously use some optimal, rational strategy for learning; for example, equilibria readily arise in competitive markets even if the individuals constituting the market do not have complete information [3], [4]. Laboratory studies of learning in decision situations have accordingly focused on how individuals integrate personal rewards and punishments. For example, the widely used Iowa gambling task (IGT) [5] requires participants to choose between card decks with different proportions of rewards and punishments. Through feedback over dozens of trials, individuals learn to choose more profitable over less profitable decks, and the rate of learning about those rewards has been linked to clinical decision-making deficits [6]–[8] and explained using computational models of decision making [9], [10].

Yet, it has long been recognized that many real-world decisions are made in a social context – choices depend not only on personal goals, but also potential benefits for others. Most laboratory studies of decision making in social contexts have used simple interactive games [11], usually involving a one-shot distribution of money between oneself and others. Results from such tasks allow researchers to calculate measures of other-regarding preferences, such as the degree to which altruism, fairness, and reciprocity modulate decisions [12]. Economists have formalized these behaviors in models – such as the social preference model [13] and social exchange theory [14], [15] – in which outcomes for others are transformed into subjective payoffs for oneself. While such interactive games have led to seminal theoretical advances in understanding of social preference, they are explicitly constructed to minimize social learning (e.g., limited numbers of trials, full information is given to participants). In contrast, everyday social decisions are often made in a dynamic environment that requires learning from past rewards. Thus, implicit measures of reward learning and preference may be more sensitive than explicit measures, both for predicting future choices and for probing psychiatric disorders associated with social dysfunction [16].

To investigate how reward learning contributes to social preferences, we designed a social gambling task (SGT) that incorporates rewards for oneself and for a desirable charity into an interactive and dynamic game. Our task is based upon the well-studied IGT. In the SGT, every draw of a card leads to a payoff for oneself and a payoff for a charity – with each of four decks having a different relative distribution of each sort of payoff. We use charitable donations as a prototypic method for eliciting social preferences: even though donations are made without offsetting material benefits to oneself [17], they reliably engage processes associated with social cognition [18], [19] and with reward processing [20], [21].

To understand how participants learned about rewards during the SGT, we adopted a reinforcement-learning modeling approach [22]. By incorporating both self and charity rewards into the model, we could probe subjects' relative sensitivity to self versus charity rewards. Converging evidence from participants' choices and modeling results shows that social learning – relatively higher weighting of charitable as opposed to personal outcomes – predicts altruistic behavior.

## Materials and Methods

### Participants

Data were collected from 103 participants (44 men, 59 women; age range  = 18–36 years, *M* = 23 years, *SD* = 4 years). We excluded one participant who chose the same deck on all 100 trials, leaving a final sample of 102 participants. All participants provided written informed consent under a protocol approved by the Duke University Institutional Review Board. The data set collected from all 103 participants will be publically available upon request.

### Procedure

Before task presentation, participants read instructions on the study paradigm and a brief introduction to a charity foundation to which earnings would be donated. As a charity with broad appeal for our participants, we used the “Make a Wish!” foundation, which helps young children with severe illness accomplish their dreams. Participants then performed the social gambling task described below. Upon completion of that task, they filled out a battery of measures that includes general reward sensitivity and other-regarding preferences: BAS subscore of the Behavioral Inhibition/Behavioral Activation System (BIS/BAS, an index of approach and avoidance tendencies) [23] and the Temporal Experience of Pleasure Scale (TEPS, an index of reward experience and anticipation) [24] as reward sensitivity measures, the Interpersonal Reactivity Index (IRI) as an assessment of dispositional empathy towards others [25] and the altruism and selfishness scores of the Helping Orientation Questionnaire (HOQ) as indices of pro-social personality [26]. The HOQ questionnaire is composed of twenty-three multiple-selection questions, such as: *“A person in one of your classes is having trouble at home and with school work. You: A) help the person as much as you can. B) tell the person not to bother you. C) leave the person alone to work out his or her own problems. D) agree to tutor the person for a reasonable fee”.* Experimental studies have indicated that greater altruistic tendencies as measured by HOQ predict greater helping of a person in need [27]. After completion of the task, we asked the participants the following questions related to the charity foundation. How much do you agree with the goal of this charity foundation? (1 =  very much disagree, 5 =  very much agree), In a scale of 1 to 5 how much did the goal/mission of the charity affect your decision in choosing the card decks? (1 =  not at all, 5 =  very much), How did you feel when you won money for yourself? (Happiness_self_: 1 =  very unhappy, 5 =  very happy), How did you feel when you won money for the charity (Happiness_charity_: 1 =  very unhappy, 5 =  very happy)? At the end of each experimental session, the experimenter paid the participant according to their choices and made a separate donation to the charity (both ranging from $8 to $24).

### Social Gambling Task (SGT)

**Figure 1:**
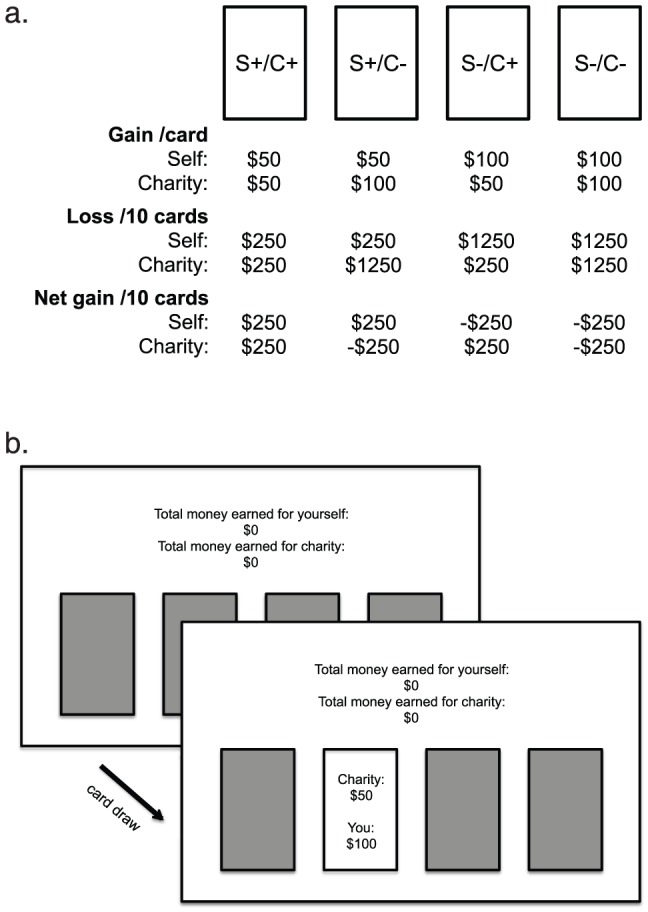
Payout structure for each card deck of the Social Gambling Task (SGT). S =  self, C =  charity, + =  gain deck, −  =  loss deck. For the gain decks, each card draw always gave $50 but in some trials it was also associated with losses ranging between $25 and $75 (i.e. loss of $25, $50 or $75). Each card draw only displayed the outcome combining the gain and loss, which ranged between −$25 to $25. Every 10 trials had 5 loss trials with a total loss of $250, thus resulting in a net gain of $250. For the loss decks, each card draw always gave $100 with some trials giving losses ranging between $150 and $350 (i.e. loss of $150, $200, $250, $300 or $350). The outcome displayed ranged between −$250 to −$50. Every 10 trials had 5 loss trials with a total loss of $1250, thus resulting in a net loss of $250. (a.) and an example screen display of a trial in SGT (b.).

Our task modifies the basic structure of the Iowa gambling task (IGT) [5], such that each card deck is associated not only with monetary outcomes for the player, but also monetary outcomes for a charity. The four decks had differing payoffs for self versus charity in a 2×2 fully orthogonal design (S+/C+, S+/C−, S−/C+, S−/C−; S =  self, C =  charity, + =  gain deck, −  =  loss deck); see Figure 1 for details on card payout structure. For the gain decks, each card draw always gave $50, but in some trials it was also associated with losses ranging between $25 and $75 (i.e. loss of either $25, $50 or $75). Each card draw only displayed the outcome combining the gain and loss which ranged between −$25 to $25. Every 10 trials had 5 loss trials with a total loss of $250, thus resulting in an accumulated net gain of $250. For the loss decks, each card draw always gave $100 with some trials giving losses ranging between $150 and $350 (i.e. loss of either $150, $200, $250, $300 or $350). The outcome displayed ranged between −$250 to −$50. Every 10 trials had 5 loss trials with a total loss of $1250, thus resulting in an accumulated net loss of $250. The outcome values were deterministic in that we used these fixed values across participants. The four card decks were horizontally displayed in the center of the screen.

After the player chose a deck, two separate payouts pertaining to self and to charity appeared. After a total of 100 selections, the task automatically stopped without warning; participants did not know how many trials the task comprised. The total accumulated amount won for self and for charity was shown on top of the screen on every trial. We gave the following instructions to the participants: “In this study you will earn money for yourself and for a charity by playing a card deck game. There will be 4 card decks shown on the screen horizontally. You will select one card at a time by pressing the corresponding four keys. Each card draw will tell you how much you won for yourself and for the charity. For both domains, sometimes you will win more money than others and sometimes you will even lose money. The total money you earned for yourself and for charity will be shown on top of the screen. You are absolutely free to switch from one deck to another at any time, and as often as you wish. The only hint we can give you, and the most important thing to note is this: Out of the four decks of cards, there are some that are worse than others for you or for charity. Also note that the computer does not change the order of the cards once you begin the game. That is, it does not make you win or lose at random.”

### Data analysis

SPSS Statistics 20.0.0 statistical software (SPSS Inc., Chicago, IL) was used for all statistical analyses. The statistical threshold for all analyses was a *p* value of .05. When computing tests for repeated measures data, the Huynh–Feldt epsilon [28] was used to determine whether data met the assumption of sphericity (Σ>0.75). In cases where the sphericity assumption was not met, the F statistic was evaluated for significance using the Huynh–Feldt adjusted degrees of freedom. The reinforcement learning model was implemented using MATLAB (The Mathworks, Natick, MA).

### Reinforcement Learning Model

To characterize the evolution of subjects' choices during the experiment, we fit individual subjects' choices with a reinforcement learning model. Decisions were assumed to be based on action values for each deck, calculated in response to the history of received rewards of both types. Specifically, the value (*V_ij_*) of deck *i* on a given trial *j* is defined as:
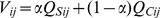
(1)where (*Q_Sij_*) and (*Q_Cij_*) are the estimated rewards to self and charity, respectively, for deck *i* on trial *j*. The parameter *α* ranges between completely self-interested valuation (*α* = 1) and completely charity-interested valuation (*α* = 0), as fit individually for each subject based on observed choice patterns. *Q_Sij_* and *Q_Cij_* are updated each time the particular deck is chosen.



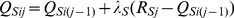
(2)

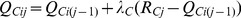
(3)


*R_S_* and *R_C_* are the observed reward outcomes for self and charity on the current trial *j*, *λ_S_* and *λ_C_* are learning rates, and (*R_j_ – Q_j-1_*) are the reward prediction errors for each reward type.

Our model links option values (*V_ij_*) with choice probabilities via the softmax rule [22]. 
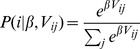



Here, P is the probability of choosing a particular deck *i* on a given trial *j* and *β* is a so-called “greediness” parameter with *β = ∞* corresponding to perfectly greedy (exploitive) choice behavior.

Option values *V_ij_* were weighted sums of separately learned self and charity values for each deck, with the proportion controlled by a parameter *α*. We fit models using a maximum log likelihood method implemented with MATLAB's constrained optimization routines (i.e. fmincon). For each subject, we fit *β*, *α*, and two learning rates that captured responses to self and charity rewards.

## Results

### Social Gambling Task

**Figure 2:**
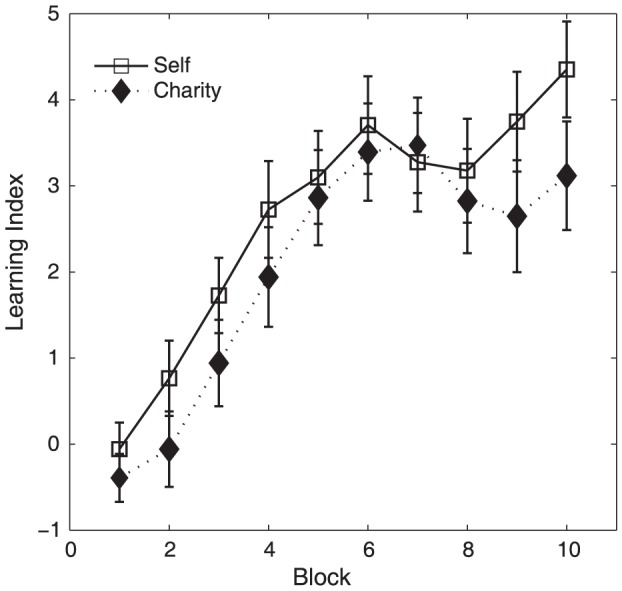
Learning index (LI) for self and charity across 10 blocks showing change in learning across time. Each block consists of 10 card draws. Learning index for self (LI_self_) and charity (LI_charity_) was independently calculated as follows: LI_self_  =  (# of cards drawn from S+/C+ and S+/C− decks) - (# of cards drawn from S−/C+ and S−/C− decks), LI_charity_  =  (# of cards drawn from S+/C+ and S−/C+ decks) - (# of cards drawn from S+/C− and S−/C− decks).

We examined the relative bias in choosing cards from gain decks compared to loss decks, for self and charity independently. To determine how learning emerged across time for self and charity, we looked at the changes in the relative proportion of choices from gain decks versus loss decks – a quantity hereafter called the Learning Index (LI) – across 10 blocks of 10 trials each. Specifically, learning index for self (LI_self_) and charity (LI_charity_) was independently calculated as follows: LI_self_  =  (# of cards drawn from S+/C+ and S+/C− decks) - (# of cards drawn from S−/C+ and S−/C− decks), LI_charity_  =  (# of cards drawn from S+/C+ and S−/C+ decks) - (# of cards drawn from S+/C− and S−/C− decks). A repeated measures ANOVA with domain (LI_self_ vs. LI_charity_) by block (block 1–10) as within-subject factors showed a significant main effect of block (F_6.18,624.36_ = 18.8, p<0.001) (Figure 2). A post hoc linear contrast for block was also significant (F_1,101_ = 50.05, p<0.001). No main effect of domain (F_1,101_ = 2.85, p = 0.095) or domain by block (F_9,909_ = 0.68, p = 0.73) interaction was found.

LI_self_ became significantly different from zero by block 3 (t_101_ = 3.95, p<0.001) but not at block 1 or 2. LI_charity_ became significantly different from zero by block 4 (t_101_ = 3.35, p<0.005), but not earlier. When examining each deck independently, the rate of choices of the S+/C+ deck increased across blocks, with participants choosing that deck beyond chance level beginning from block 3 (t_101_ = 3.54, p<0.001). These results indicate that subjects, on average, learned reward contingencies for both domains; however, learning occurred approximately one block earlier for self rewards.

**Figure 3:**
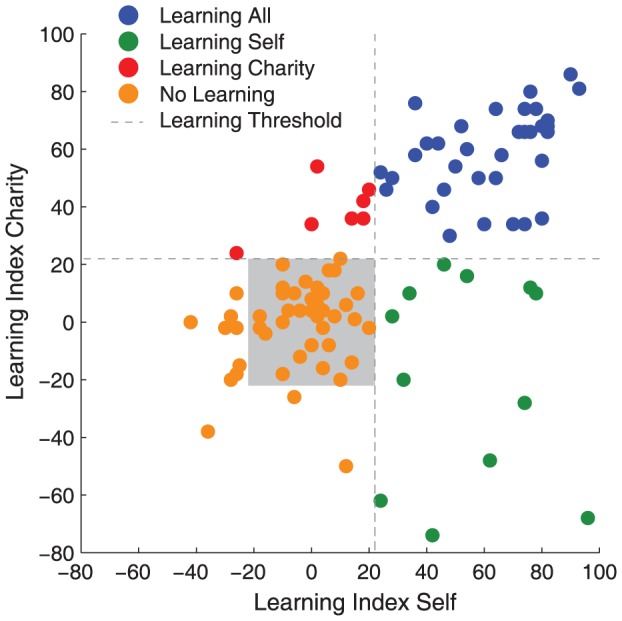
Individual difference in learning index (LI) for self versus charity. Each data point indicates an individual participant's LI values for self and charity. Learners for both self and charity are shown in blue, learners only for self in green and learners only for charity in red. Data points within the gray box area represent the non-learners. Orange data points outside the gray box represent anti-learners (i.e., participants who systematically chose the loss decks in one or both domains).

**Figure 4:**
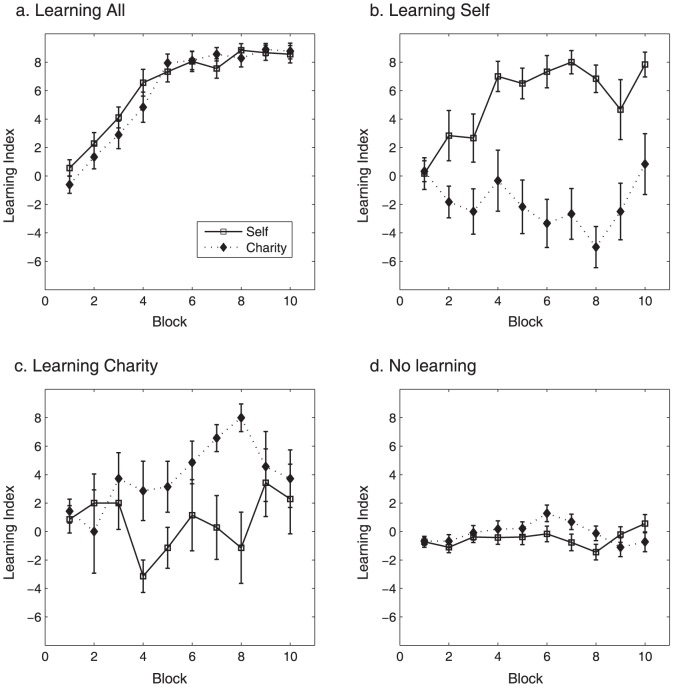
Learning index for self (open square) and charity (filled diamond) across 10 blocks in the subgroups. Each subplot shows the average across participants from each subgroup as follows: Learning All (a.), Learning Self (b.), Learning Charity (c.) and No learning (d.).

We next categorized individuals into learners and non-learners for each domain (Figure 3 and 4). For the purpose of categorization, we used the relative proportion of choices of the gain decks compared to the loss decks. Following this criterion, we defined learners as individuals who chose decks involving gains significantly more than decks involving losses, throughout the entire experiment (LI>22, based on binomial test at *p*<0.05). This led to the following subgroups: learners in both the self and charity (Learning All group, N = 36), learners for only self but not the charity (Learning Self group, N = 12), learners for only charity but not the self (Learning Charity group, N = 7), those who chose the loss decks significantly more than the gain decks in one or both the self and charity domain (Anti-Learning group, N = 11), and individuals who did not show evidence of learning for either deck (Non-Learning group, N = 36). For subsequent analyses of individual differences, we excluded the Non-Learning group because those individuals' choices could not be explained by our reinforcement-learning model (see results for reinforcement learning model below).

### SGT performance and other-regarding behavior

Next, we evaluated whether individual differences in reward learning – specifically, the difference between LI_charity_ and LI_self_ – predicted self-reported other-regarding preferences. Significant correlation was observed between the LI difference and the altruism score of HOQ (*r* = 0.33, *p* = 0.006), such that more altruistic participants showed relatively greater learning for charity than learning for self. LI_charity_ alone also correlated with altruism (*r* = 0.33, *p* = 0.008), but LI_self_ did not (*r* = −0.012, *p*>0.9). These results indicate that learning for others correlated with one's altruistic tendencies, even after controlling for overall learning as measured by LI_self_. LI_charity_ alone also correlated with HOQ selfishness subscore (r = −0.31, p = 0.011). The relationships we observed between learning indices and other-regarding behavior were still present but less significant when including all participants including the non-learners (LI difference score & altruism: r = 0.23, p = 0.023, LI_charity_ & altruism: r = 0.21, p = 0.036, LI_charity_ & selfishness: r = −0.24, p = 0.014). We additionally explored whether there was a relationship between SGT performance and general reward sensitivity. We found no relationship between either the BAS or TEPS score and the LI difference or the LI_self_ or LI_charity_ alone (all p values >0.1).

We also looked at the relationship between their feelings towards the charity foundation assessed by the additional questions we asked about the charity foundation and the learning index difference (LI_charity_ – LI_self_). We found that the degree to which the goal/mission of the charity affected one's decision correlated with learning index difference (r = 0.387, p<0.0001) such that the more the charity goal affected the decision the better they learned for charity relative to self. The happiness rating difference (i.e. Happyness_charity_ - Happyness_self_) also correlated with learning index difference (r = 0.241, p = 0.015), such that the more they were happy earning rewards for charity as compared to self, the more better they learned for charity relative to self.

### Reinforcement learning model of the SGT

To further quantify processes underlying participants' behavior, we fit a reinforcement learning model to each individual's choices in the task. We assessed model performance by calculating the percentage of correctly predicted choices as follows. On each trial, we considered the deck with the highest probability of being chosen as the model's prediction. We used the probability determined by the softmax rule as described in the Methods section. Trials on which the subject chose that option were counted as correct. In trials in which there were two or three options tied in value, subject's choice of one of these tied values was considered correct. Trials with all four options tied were considered as incorrect trials.

**Figure 5:**
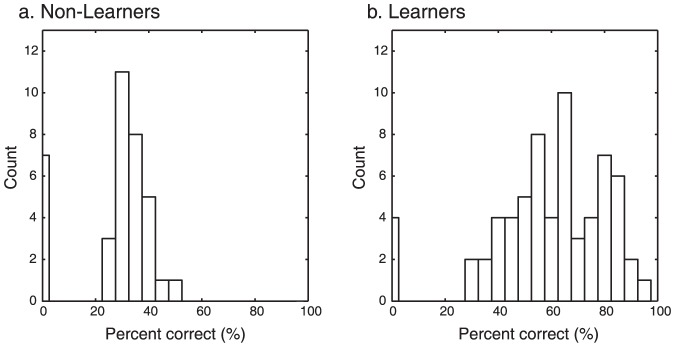
Histograms showing the percentage of accurately predicted choices across all trials in the non-learners (a.) and the learners (b.).

The model predicted choice above chance level (25%, one out of four decks) in a majority of participants (90 out of 102). However, in 12 individuals, model predictions proved poor, with predictive accuracy below chance. Unsurprisingly, this group overlapped significantly (8 out of 12) with those subjects in the non-learners who failed to display stable preferences and showed random card selection over the course of the experiment (LI between −22 and 22 in both self and charity domain). To verify this, we examined the proportion of accurately predicted choices across all trials (percent match) separately in the non-learners showing random card selection and the rest of the participants (learners). We found that most non-learners had fewer than 50% of choices accurately predicted (median near 35%), while most learners fell above 50% (Figure 5). Four subjects showed 0% match between model prediction and actual choice despite the fact that their choices were not random. These people however, consistently chose S−/C− more than any other decks despite negative outcomes, behavior that cannot be captured by a model such as ours, which assumes correct learning (option values converge to expected values) and rational choice (decks with higher values should be chosen more often). Excluding these four individuals did not change the result of the correlation analysis between the model estimate α and altruism score the main result of which is described below (r = −0.29, p = 0.021).

**Figure 6:**
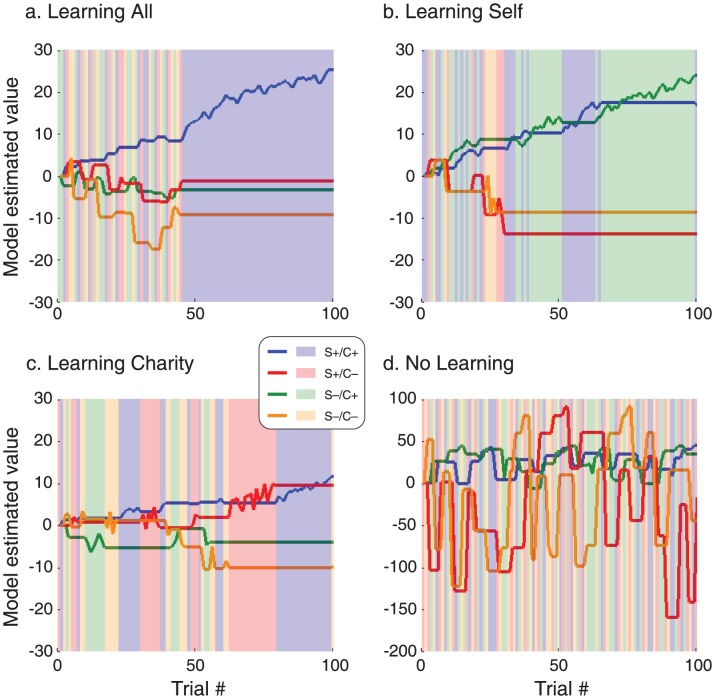
Value of each deck estimated by the reinforcement learning model across 100 trials. Each plot shows an example of value change in the four decks in each of the subgroups: Learning All (a.), Learning Self (b.), Learning Charity (c.) and No learning (d.). Color schemes for each deck are as follows: blue  = S+/C+, green  = S+/C−, red  = S−/C+, orange  = S−/C−. The background of each plot demonstrates the decks chosen by the subject on each trial following the same color schemes.

Within our model, an *α* near 1 indicates that subjects chose almost entirely based on rewards to themselves, while an *α* near 0 indicates that subjects focused solely on charitable outcomes. The parameter *α* was correlated with both the LI_self_ (r = 0.38, p = 0.002) and LI_charity_ (r = −0.32, p = 0.01) suggesting that the relative weighting of self versus charity reward on each trial is associated with overall choice behavior. Finally, each model includes a parameter that captures the variability of subjects' choices; i.e., their tendency to explore suboptimal alternatives. Figure 6 shows prototypical examples of learning across trials. Subjects with *α* near 1 exhibited more choices of S+/C+ and S+/C− deck, while subjects with *α* near 0 chose the S+/C+ and S−/C+ decks more often. In addition, subjects with low learning rates exhibit slow, measured change in the valuation of each deck, while subjects with high learning rates exhibited rapid alterations in value following unexpected outcomes. In addition, some subjects exhibit no learning, with choices exhibiting no clear preference over the course of the experiment (See Table 1 for summaries on subgroup results.).

**Table 1:** Summary of subgroup statistics.

Choice				
	S+/C+	S+/C−	S−/C+	S−/C−
Learning All	68.9±11.9	12.3±6.5	10.6±7.4	8.2±3.4
Learning Self	30.7±16.5	46.3±18.5	9.8±5.0	13.3±8.3
Learning Charity	41.1±7.0	12.1±2.5	28.3±5.7	18.4±5.9
No Learning	24.2±7.9	23.2±7.0	25.2±5.6	27.3±6.2
Total	41.9±23.0	21.3±13.6	18.4±9.9	18.3±10.4

Reinforcement learning model					
	Model performance (percent correct)	Exploit/Greediness (β)	Self/Charity reward sensitivity (α = 1, purely selfish; α = 0, purely charitable)	Learning rate self (λs)	Learning rate charity (λc)
Learning All	72.81±11.79	0.09±0.02	0.50±0.30	0.20±0.26	0.27±0.38
Learning Self	55.33±13.34	0.07±0.04	0.88±0.26	0.17±0.28	0.38±0.43
Learning Charity	49.29±8.90	0.06±0.04	0.13±0.10	0.91±0.22	0.15±0.19
No Learning	27.13±16.27	−	−	−	−
Total	48.09±24.93	0.08±0.03	0.54±0.35	0.29±0.35	0.28±0.37

The first four columns of the upper panel show mean ±S.D of the number of cards selected for each deck. The No learning group includes both the anti-learners and non-learners. Model parameter statistics for No Learning group is excluded due to close to chance level accuracy (around 25%) in predicting choice.

We compared the learning rate for self and for charity in the subgroups using a repeated measure ANOVA with reward domain (self vs. charity) as within subject factors and subgroup (Learning All vs. Learning Self vs. Learning Charity. No Learning subgroup was excluded due to unsuccessful model fitting in group.) as between subject factors. We found a significant main effect of subgroup (F_2,52_ = 6.33, p = 0.003) and domain by subgroup interaction (F_2,52_ = 10.56, p<0.0001). A follow up test comparing learning rates across pairs of subgroups showed that average learning rate was significantly different between “Learning Self” and “Learning Charity” subgroups (F_1,17_ = 4.52, p = 0.048) and between “Learning All” and “Learning Charity” subgroups (F_1,41_ = 4.52, p = 0.001). Pairwise comparisons of the two learning rates in each subgroup showed a significant difference between the two in the “Learning Charity” subgroup (t_6_ = 8.53, p<0.0001) with greater learning rate for self than charity and a marginally significant difference between the two in the “Learning Self” subgroup (t_11_ = −2.104, p = 0.059) with greater learning rate for charity than self. These results suggest that efficient learning expressed as significantly greater gain decks chosen compared to loss decks is better achieved by slow incremental change in valuation of decks (as represented by small learning rates) as opposed to abrupt alterations in valuation (as represented by large learning rates).

We tested whether parameters in the learning model predicted other-regarding tendencies. There was a significant negative relationship between α and the altruism subscore of HOQ (r = −0.29, p = 0.017), demonstrating that the relative learning bias toward charitable rewards in the SGT predicted greater altruistic behavior in the HOQ.

We also compared our original model with two other models as belows to test whether the original model gave the best model fit.model 1 (original):



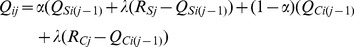
model 2 (only considers self reward):



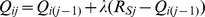
model 3 (only considers charity reward):



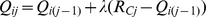



We used the Akaike information criterion (AIC, [29]) as below to assess the quality of the models. 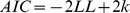
k is the number of parameters and LL is the log likelihood [30] value. AICs for the three models were computed for each individual. The model with the minimum AIC value is most preferred model [29].

- Out of 102 total subjects, model 1 gave the minimum AIC value in 57 subjects, model 2 gave the minimum AIC value in 25 subjects and model 3 gave the minimum AIC value in 24 subjects.
- Out of 66 subjects excluding the non-learning group, model 1 gave the minimum AIC value in 54 subjects, model 2 gave the minimum AIC value in 6 subjects and model 3 gave the minimum AIC value in 9 subjects.
- Within the two subgroups, Learning Self and Learning Charity, model 1 gave the minimum AIC value in the majority of participants (83% in Learning Self and 86% in Learning Charity subgroups).

Based on these additional analyses, we believe that our current model including both the self and charity reward into consideration provides the best model of performance. We should also note that, in several cases where the smaller models (self-only or charity-only) provided better fits, this coincides with a fit of model 1 in which alpha was very near 0 or 1. That is, the smaller models are better fits precisely when model 1 would also reduce to them.

## Discussion

Our task, designed to characterize decision-making behavior in a social context, relies on three key features. First, the card decks capture real-life tradeoffs between benefits to oneself and benefits to others. Second, decisions are made in a dynamic environment that involves uncertainty. Third, preferences emerge over the course of the task, unlike conventional single-shot games.

Previous studies using interactive games have shown that there are significant individual differences in the expression of pro-social behaviors like altruism, fairness and trust [13], [31], [32], with various psychological and neural factors linked to that heterogeneity [18], [33], [34]. Our results demonstrate that heterogeneity also exists in the learning of pro-social reward contingencies: the individuals with greater altruistic behavior as measured by the Helping Orientation Questionnaire (HOQ) [26] had relatively greater learning (i.e. larger learning index, that is greater difference in the number of gain decks chosen versus loss decks chosen) for charity compared to self. This result supports the idea that an individual's pro-social tendencies are associated with their abilities to acquire and use of information about others' outcomes, in order to make better pro-social decisions. We note that the results only show an association between altruistic tendencies and pro-social reward learning in our task; thus, we cannot determine whether altruistic tendencies drive pro-social learning or the learning results in altruistic tendencies. Additionally the correlation between the two measures may also be driven by other mediating factors such as one's desire to appear altruistic in the self-report measure.

While we found significant relationship between pro-social learning and altruism as measured by HOQ, we did not find a relationship with empathy score measured by IRI. HOQ and IRI were independently included in this study to measure altruism and empathy which are considered to be two separate entities. This is supported by a non-significant correlation between the two measures (r = 0.15, p = 0.15). Previous literature have shown that empathy may increase altruistic motivation, but that empathy does not necessarily drive altruistic behavior [35]–[38]. Our results showing only a significant relationship between pro-social learning and altruism but not with empathy also suggests that feeling of empathy may not be sufficient enough to drive greater reward sensitivity for charity than self.

We also did not find any relationship between LI_self and the BAS and TEPS scores. These measures have been used as a measure of emotional reactivity to rewards [39]–[43]. BIS/BAS has previously shown to predict original IGT performance [44], [45]. Our task differs from the IGT in that there are two reward outcomes to consider: self and charity. Thus learning may not be purely driven by motivation to increase one's own payout but also to benefit others, which may not be captured by these scales essentially centered on one's own reward experience.

It is worth noting however that the choices on decks may not necessarily reflect learning *per se*, but perhaps a preference toward the particular deck. For example, one might actively select the non-charitable decks even if they have learned and had the knowledge about the decks because they did not value giving money to the charity. We cannot exclude the possibility that some participants have accurate information about the consequences of the decks, but chose not to adjust their behavior based on that information. We did observe, however, that the degree to which participants agreed with the goal of the charity did not correlate with learning indices, but the degree to which they perceived the charity goal to influence their decision was correlated. This suggests that disagreement with the goals of the charity did not drive results in our experiment.

An important aspect of our study lies in using a reinforcement-learning model to understand the process underlying SGT performance. These models have been repeatedly applied to reward-based learning [46], [47] as well as social interactions [48], [49]. While learning index (LI) is an aggregate measure of learning reflecting individual's understanding of the decks' reward contingencies and thus can serve as a summary measure of performance across the whole task, it is possible that this measure may capture aspects of preference rather than learning *per se* as described in the previous paragraph. The reinforcement learning model on the other hand gives an estimate of how participants adjust their behavior based on the reward outcomes on a trial by trial basis. Thus the parameters from reinforcement learning model gives a more accurate estimate of learning taking into consideration the trial by trial changes in behavior throughout the task. In other words, the modeling allows us to understand the mechanisms underlying the choice behaviors during SGT that involves taking into considerations both self and charity reward outcomes.

Our model estimates each individual's relative sensitivity to rewards for self relative to rewards for charity, as expressed in the parameter α. Our model reliably tracked the choice behavior of individuals across a range of choice preferences, and its sensitivity parameter was correlated with self-reported altruism. This may be because those with stronger other-regarding preferences are more attentive to the outcomes of others, and attention to outcomes modulates learning [50], [51]. Additionally, our model classifies individuals based on their reward learning – in two dissociable domains – and separates selfish and other-regarding behavior over the learning process. These results are consistent with the idea that social learning can co-opt more basic systems for learning about one's own rewards, an assumption of models of vicarious reward [52], [53] which is also supported by recent neuroimaging findings showing overlapping brain mechanisms that process rewards directed to self and to others [54]–[56].

In summary, we developed a social gambling paradigm in which one learns to make advantageous decisions for others as well as for oneself. We have shown that people readily learn about others' reward contingencies, just as they do for their own rewards – although learning is less rapid for social rewards. Furthermore, the relatively greater valuation for social rewards compared to self rewards was associated with self-reported pro-social tendencies, as supported by converging evidence showing that self-reported altruism correlates with both the LI difference score and the model parameter α. These results indicate that one's implicit pro-social motivations interact with one's capability of considering both one's own and others' rewards when making decisions.

## References

[1] Becker GS (1976) The Economic Approach to Human Behavior. Chicago: University of Chicago Press.

[2] Smith A (1776) The Wealth of Nations. London: W. Strahan & T. Cadell.

[3] Smith VL (1962) An experimental study of competitive market behavior. Journal of Political Economy 70: 111–137.

[4] Smith VL (1982) Microeconomic systems as an experimental science. American Economic Review 72: 923–955.

[5] Bechara A, Damasio AR, Damasio H, Anderson SW (1994) Insensitivity to future consequences following damage to human prefrontal cortex. Cognition 50: 7–15. 8039375 10.1016/0010-0277(94)90018-3

[6] Brand M, Kalbe E, Labudda K, Fujiwara E, Kessler J, et al (2005) Decision-making impairments in patients with pathological gambling. Psychiatry Res 133: 91–99. 15698681 10.1016/j.psychres.2004.10.003

[7] Brogan A, Hevey D, Pignatti R (2010) Anorexia, bulimia, and obesity: shared decision making deficits on the Iowa Gambling Task (IGT). J Int Neuropsychol Soc 16: 711–715. 20406532 10.1017/S1355617710000354

[8] Poletti M, Cavedini P, Bonuccelli U (2011) Iowa gambling task in Parkinson's disease. J Clin Exp Neuropsychol 33: 395–409. 21140314 10.1080/13803395.2010.524150

[9] Busemeyer JR, Stout JC (2002) A contribution of cognitive decision models to clinical assessment: decomposing performance on the Bechara gambling task. Psychol Assess 14: 253–262. 12214432 10.1037//1040-3590.14.3.253

[10] Yechiam E, Busemeyer JR, Stout JC, Bechara A (2005) Using cognitive models to map relations between neuropsychological disorders and human decision-making deficits. Psychol Sci 16: 973–978. 16313662 10.1111/j.1467-9280.2005.01646.x

[11] Rilling JK, Sanfey AG (2011) The neuroscience of social decision-making. Annu Rev Psychol 62: 23–48. 20822437 10.1146/annurev.psych.121208.131647

[12] Fehr E (2008) Social preference and the brain. In: P. W Glimcher, E Fehr, C Camerer and R. A Poldrack, editors.Neuroeconomics: Decision making and the brain.New York: Academic Press. pp. 215–232.

[13] Fehr E, Schmidt K (2003) Theories of fairness and reciprocity: evidence and economic applications. In: M Dewatripont, L. P Hansen and S. J Turnovsky, editors.Advances in Economics and Econometrics.Cambridge: Cambridge University Press. pp. 208–257.

[14] Homans GC (1958) Social behavior as exchange. American Journal of Sociology 63: 597–606.

[15] Thibaut JW, H.H K (1959) The social psychology of groups. New York: Wiley.

[16] Montague PR, Dolan RJ, Friston KJ, Dayan P (2012) Computational psychiatry. Trends Cogn Sci 16: 72–80. 22177032 10.1016/j.tics.2011.11.018 PMC3556822

[17] Yokum D, Rossi F (2009) A neurocognitive perspective on charitable giving.

[18] Tankersley D, Stowe CJ, Huettel SA (2007) Altruism is associated with an increased neural response to agency. Nat Neurosci 10: 150–151. 17237779 10.1038/nn1833

[19] Zaki J, Mitchell JP (2011) Equitable decision making is associated with neural markers of intrinsic value. Proc Natl Acad Sci U S A 108: 19761–19766. 22106300 10.1073/pnas.1112324108 PMC3241792

[20] Harbaugh WT, Mayr U, Burghart DR (2007) Neural responses to taxation and voluntary giving reveal motives for charitable donations. Science 316: 1622–1625. 17569866 10.1126/science.1140738

[21] Moll J, Krueger F, Zahn R, Pardini M, de Oliveira-Souza R, et al (2006) Human fronto-mesolimbic networks guide decisions about charitable donation. Proc Natl Acad Sci U S A 103: 15623–15628. 17030808 10.1073/pnas.0604475103 PMC1622872

[22] Sutton RS, Barto AG (1998) Reinforcement Learning: An Introduction. Cambridge, MA: MIT Press.

[23] Carver CS, White TL (1994) Behavioral-Inhibition, Behavioral Activation, and Affective Responses to Impending Reward and Punishment - the Bis Bas Scales. J Pers Soc Psychol 67: 319–333.

[24] Gard DE, Gard MG, Kring AM, John OP (2006) Anticipatory and consummatory components of the experience of pleasure: A scale development study. J Res Pers 40: 1086–1102.

[25] Davis MH (1983) Measuring Individual-Differences in Empathy - Evidence for a Multidimensional Approach. J Pers Soc Psychol 44: 113–126.

[26] Romer D, Gruder CL, Lizzadro T (1986) A Person Situation Approach to Altruistic Behavior. J Pers Soc Psychol 51: 1001–1012.

[27] Carlo G, Eisenberg N, Troyer D, Switzer G, Speer AL (1991) The Altruistic Personality - in What Contexts Is It Apparent. J Pers Soc Psychol 61: 450–458. 1941516 10.1037//0022-3514.61.3.450

[28] Huynh H, Feldt LS (1970) Conditions under which mean square ratios in repeated measurement designs have exact F-distributions. Journal of the Americal Statistical Association 65: 1582–1589.

[29] Akaike H (1974) A new look at the statistical model identification. IEEE Transactions on Autonomic Control 19: 716–723.

[30] Edwards AWF (1972) Likelihood. Cambridge University Press.

[31] Kishida KT, King-Casas B, Montague PR (2010) Neuroeconomic approaches to mental disorders. Neuron 67: 543–554. 20797532 10.1016/j.neuron.2010.07.021 PMC2957178

[32] Murphy RO, Ackermann KA, Handgraaf MJJ (2011) Measuring Social Value Orientation. Judgm Decis Mak 6: 771–781.

[33] Donaldson ZR, Young LJ (2008) Oxytocin, Vasopressin, and the Neurogenetics of Sociality. Science 322: 900–904. 18988842 10.1126/science.1158668

[34] Morishima Y, Schunk D, Bruhin A, Ruff CC, Fehr E (2012) Linking Brain Structure and Activation in Temporoparietal Junction to Explain the Neurobiology of Human Altruism. Neuron 75: 73–79. 22794262 10.1016/j.neuron.2012.05.021

[35] Archer RL, Diaz-Loving R, Gollwitzer PM, Davis MH, Foushee HC (1981) The rold of dispositional empathy and social evaluation in the empathic mediation of helping. J Pers Soc Psychol 40: 786–796.

[36] Cialdini RB, Schaller M, Houlihan D, Arps K, Fultz J, et al (1987) Empathy-based helping: is it selflessly or selfishly motivated? J Pers Soc Psychol 52: 749–758. 3572736 10.1037//0022-3514.52.4.749

[37] Fultz J, Batson CD, Fortenbach VA, McCarthy PM, Varney LL (1986) Social evaluation and the empathy-altruism hypothesis. J Pers Soc Psychol 50: 761–769. 3712222 10.1037//0022-3514.50.4.761

[38] Hauser DJ, Preston SD, Stansfield RB (2013) Altruism in the Wild: When Affiliative Motives to Help Positive People Overtake Empathic Motives to Help the Distressed. Journal of experimental psychology General. 10.1037/a0035464 24364686

[39] Beaver JD, Lawrence AD, Van Ditzhuijzen J, Davis MH, Woods A, et al (2006) Individual differences in reward drive predict neural responses to images of food. Journal of Neuroscience 26: 5160–5166. 16687507 10.1523/JNEUROSCI.0350-06.2006 PMC6674259

[40] Carter RM, Macinnes JJ, Huettel SA, Adcock RA (2009) Activation in the VTA and nucleus accumbens increases in anticipation of both gains and losses. Front Behav Neurosci 3: 21. 19753142 10.3389/neuro.08.021.2009 PMC2742668

[41] Chan RCK, Wang Y, Huang J, Shi YF, Wang Y, et al (2010) Anticipatory and consummatory components of the experience of pleasure in schizophrenia: Cross-cultural validation and extension. Psychiatry Res 175: 181–183. 19963276 10.1016/j.psychres.2009.01.020

[42] Franken IHA, Muris P (2006) BIS/BAS personality characteristics and college students' substance use. Pers Indiv Differ 40: 1497–1503.

[43] Putman P, Hermans E, van Honk J (2004) Emotional Stroop performance for masked angry faces: It's BAS, not BIS. Emotion 4: 305–311. 15456399 10.1037/1528-3542.4.3.305

[44] Desmeules R, Bechara A, Dube L (2008) Subjective valuation and asymmetrical motivational systems: Implications of scope insensitivity for decision making. J Behav Decis Making 21: 211–224.

[45] Franken IHA, Muris P (2005) Individual differences in decision-making. Pers Indiv Differ 39: 991–998.

[46] Barraclough DJ, Conroy ML, Lee D (2004) Prefrontal cortex and decision making in a mixed-strategy game. Nat Neurosci 7: 404–410. 15004564 10.1038/nn1209

[47] Daw ND, O'Doherty JP, Dayan P, Seymour B, Dolan RJ (2006) Cortical substrates for exploratory decisions in humans. Nature 441: 876–879. 16778890 10.1038/nature04766 PMC2635947

[48] Camerer C, Ho TH (1999) Experienced-Weighted Attraction Learning in Normal Form Games. Econometrica 67: 827–874.

[49] Zhu L, Mathewson KE, Hsu M (2012) Dissociable neural representations of reinforcement and belief prediction errors underlie strategic learning. Proc Natl Acad Sci U S A 109: 1419–1424. 22307594 10.1073/pnas.1116783109 PMC3277161

[50] Pearce JM, Hall G (1980) A Model for Pavlovian Learning: Variations in the Effectiveness of Conditioned But Not of Unconditioned Stimuli Psychological Review. 87: 532–552. 7443916

[51] Roesch MR, Calu DJ, Esber GR, Schoenbaum G (2010) Neural correlates of variations in event processing during learning in basolateral amygdala. J Neurosci 30: 2464–2471. 20164330 10.1523/JNEUROSCI.5781-09.2010 PMC2838173

[52] Hayden BY, Pearson JM, Platt ML (2009) Fictive reward signals in the anterior cingulate cortex. Science 324: 948–950. 19443783 10.1126/science.1168488 PMC3096846

[53] Lohrenz T, McCabe K, Camerer CF, Montague PR (2007) Neural signature of fictive learning signals in a sequential investment task. Proc Natl Acad Sci U S A 104: 9493–9498. 17519340 10.1073/pnas.0608842104 PMC1876162

[54] Behrens TE, Hunt LT, Woolrich MW, Rushworth MF (2008) Associative learning of social value. Nature 456: 245–249. 19005555 10.1038/nature07538 PMC2605577

[55] Janowski V, Camerer C, Rangel A (2013) Empathic choice involves vmPFC value signals that are modulated by social processing implemented in IPL. Soc Cogn Affect Neurosci 8: 201–208. 22349798 10.1093/scan/nsr086 PMC3575723

[56] Nicolle A, Klein-Flugge MC, Hunt LT, Vlaev I, Dolan RJ, et al (2012) An agent independent axis for executed and modeled choice in medial prefrontal cortex. Neuron 75: 1114–1121. 22998878 10.1016/j.neuron.2012.07.023 PMC3458212

